# Electrophysiological Methods for Recording Synaptic Potentials from the NMJ of Drosophila Larvae

**DOI:** 10.3791/1109

**Published:** 2009-02-06

**Authors:** Wendy Imlach, Brian D. McCabe

**Affiliations:** Department of Physiology and Cellular Biophysics, Columbia University College of Physicians and Surgeons

## Abstract

In this video, we describe the electrophysiological methods for recording synaptic transmission at the neuromuscular junction (NMJ) of Drosophila larva. The larval neuromuscular system is a model synapse for the study of synaptic physiology and neurotransmission, and is a valuable research tool that has defined genetics and is accessible to experimental manipulation. Larvae can be dissected to expose the body wall musculature, central nervous system, and peripheral nerves. The muscles of Drosophila and their innervation pattern are well characterized and muscles are easy to access for intracellular recording. Individual muscles can be identified by their location and orientation within the 8 abdominal segments, each with 30 muscles arranged in a pattern that is repeated in segments A2 - A7. Dissected drosophila larvae are thin and individual muscles and bundles of motor neuron axons can be visualized by transillumination^1^. Transgenic constructs can be used to label target cells for visual identification or for manipulating gene products in specific tissues. In larvae, excitatory junction potentials (EJP’s) are generated in response to vesicular release of glutamate from the motoneurons at the synapse. In dissected larvae, the EJP can be recorded in the muscle with an intracellular electrode. Action potentials can be artificially evoked in motor neurons that have been cut posterior to the ventral ganglion, drawn into a glass pipette by gentle suction and stimulated with an electrode. These motor neurons have distinct firing thresholds when stimulated, and when they fire simultaneously, they generate a response in the muscle. Signals transmitted across the NMJ synapse can be recorded in the muscles that the motor neurons innervate. The EJP’s and minature excitatory junction potentials (mEJP’s) are seen as changes in membrane potential. Electrophysiological responses are recorded at room temperature in modified minimal hemolymph-like solution^2^ (HL3) that contains 5 mM Mg^2+^ and 1.5 mM Ca^2+^. Changes in the amplitude of evoked EJP’s can indicate differences in synaptic function and structure. Digitized recordings are analyzed for EJP amplitude, mEJP frequency and amplitude, and quantal content.

**Figure:**
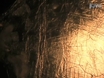


## Protocol

### Before starting prepare:

- Wandering yhird instar Drosophila larvae
- HL3.1 (Modified hemolymph-like) solution
- Sylgard (transparent silicone rubber) dissection plates prepared in small (35 x 10 mm) plastic petri dishes using methods described by Brent and McCabe (2008)^3^.
- Cut dissection pins short
- Stimulating electrode pipettes
- Sharp recording pipettes

### HL3 Solution:

1. During dissections and electrophysiological experiments, larva are immersed in HL3.1 solution^2^ that contains (in mM): 70 NaCl, 5 KCl, 4 MgCl_2_, 10 NaHCO_3_, 5 trehalose, 115 sucrose, and 5 HEPES, pH 7.2.
2. Concentrations of CaCl_2_ are added to the HL3.1 solution to provide the required concentration of extracellular Ca^2+^.
  - Larval dissections are performed at low Ca^2+^ concentrations (to avoid muscle contraction) using HL3.1 + 0.25 mM CaCl_2_, kept on ice.
  - Electrophysiological experiments are typically recorded in HL3.1 + 1 mM CaCl_2_, but this can be adjusted from 0.4 – 1 mM Ca^2+^ as required.
3. The HL3.1 solution is filter sterilized before use, and stored at 4˚C.

### Preparation of stimulating and recording pipettes:

1. Recording and stimulating pipettes are pulled with a Sutter P-2000 Laser based micropipette puller using the following settings: Heat = 390, Filament = 4, Velocity = 35, Delay = 200 and Pull = 0.
2. Stimulating electrodes are prepared from thin-walled borosilicate glass capillaries with wide ends that were slightly bigger than the width of the severed motor axons. The shaped ends are firepolished to give a smooth finish (using a Narashiga pipette polisher), to minimize damage to the nerve. Stimulating electrodes are filled with bath solution (HL3.1 + 1 mM Ca^2+^).
3. The recording electrodes are prepared from 1.2mm borosilicate glass, which is pulled to form a sharp pipette (30–60 mΩ), and filled with 3M KCl.

### Part 1: Dissection of Drosophila Larvae

1. Third instar wandering larvae are dissected to expose the muscles in the body wall as described previously^4^.
2. Dissections are performed in HL3.1 + 0.25 mM Ca^2+^ on small silicone plates (35 x 10 mm). HL3.1 solution must be ice-cold for dissections in order to anaesthetize the larva, and is kept on ice before and during dissections.
3. Larvae are dissected using methods described by Brent and McCabe (2008), that is modified for electrophysiology experiments by adding 0.25 mM Ca^2+^ to the HL3.1 dissection solution, and using short dissection pins (~2 mm in length) that are less likely to hit the microscope objective and electrodes during an experiment.
4. Following dissection, the motor axons of the larvae are severed. This is done by gently holding the CNS and raising it slightly so the peripheral nerves that innervate the muscles posterior to the ventral ganglion can be cut without damaging the muscles (Figure 1). The brain is then removed.
5. The dissected larval preparation is washed twice with HL3.1 + 1 mM Ca^2+^.

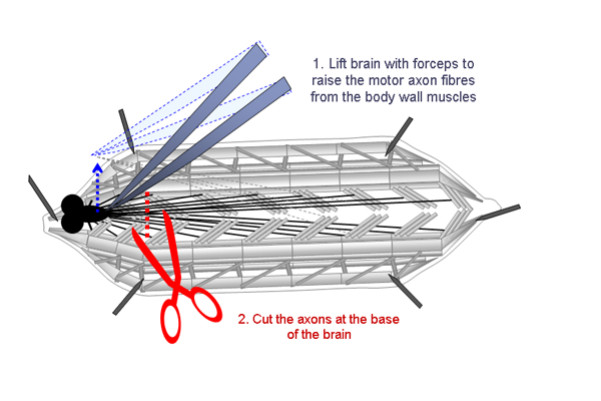
 **Figure 1.** Schematic showing the steps involved in cutting the motor axons of dissected larvae. The CNS is lifted with forceps and the motor axons are cut at the base of the brain.

### Part 2: Intracellular recordings from larval muscle cells.

1. Place the dissection plate on the electrophysiology microscope and submerge the prep with HL3.1 + 1.5 mM Ca^2+^ and fix the bath electrode so it is in contact with the solution.
2. All experiments are performed on muscle 6 within the third abdominal segment (A3). Peripheral nerves that innervate the muscles are stimulated using a suction electrode^5^.
3. Position the stimulating electrode in the dish first to avoid vibrations when the intracellular electrode is in place. Place the stimulating electrode in close vicinity to the motor neuron innervating muscle 6 and apply gentle suction until the cut nerve is inside the glass pipette. Be careful not to stretch the nerves when suction is applied. Raise the pipette slightly so it is not touching the muscle and position it so it is not pulling on the cut nerve fibers (Figure 2). 
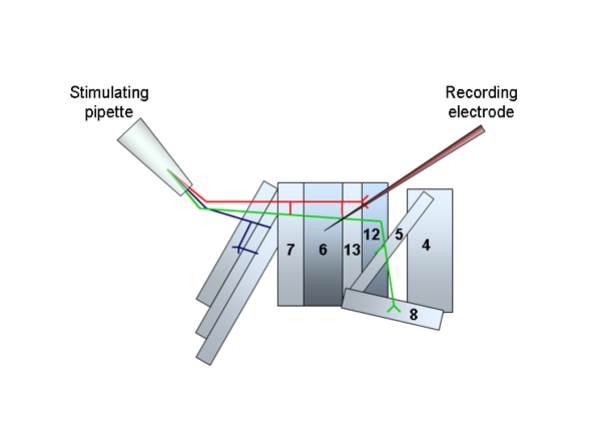
**Figure 2.** A schematic of the musculature and innervating motor neurons of one abdominal segment from the Drosophila larva. The position of the stimulating pipette and severed nerves and the recording electrode in position at muscle 6 are illustrated.
4. Intracellular recordings are made with sharp microelectrodes filled with 3 M KCl. Position the recording electrode above the centre of muscle 6 on the third abdominal segment (A3) and adjust the input offset so it reads zero for the bath solution. Slowly lower the electrode and approach the muscle under high optical magnification until it touches the muscle surface. Watch the oscilloscope to confirm that the muscle has been penetrated. The resting membrane potential should be at least -60 mV, or the animal should not be used. Sporadic miniature endplate potentials should now be visible. Leave the cell to stabilize for one minute before starting to record.
5. Changes in membrane potential are detected with an Axon HS-2A head stage and an Axoclamp 2B amplifier and recorded with Clampex v 8.2.0.235 (Axon Instruments). The Axoclamp 2B is interfaced to a computer that runs pCLAMP software and works in conjunction with the Digidata 1322A interface.
6. Cells were recorded for 3 minutes without any stimuli to measure mEJP responses. Traces were analysed using Mini analysis software (Synaptosoft, v 6.0.3) and the mEJP amplitude and frequency determined.

### Nerve Stimulation:

1. The severed end of the motor neuron is stimulated with a series of square voltage pulses (0.3 ms duration) at an intensity that is sufficient for both motor axons, to evoke consistent responses in the whole muscle.
2. The stimulus is generated with the Master-8 pulse generator, which is programmed to deliver pulses continuously according to the duration and interval times.
3. Allow five seconds between stimuli for synaptic recovery at the NMJ.
4. When determining the strength of the stimulus, begin with the lowest level and increase the intensity slowly over several trials. The minimum stimulus intensity that results in an evoked response should be used in experiments.
5. When the appropriate stimulus intensity is reached, there should be one compound EJP and one muscle contraction evoked by the stimulus.
6. Record at least 10 evoked potentials from each muscle and average the amplitudes.

### Part 3: Representative Results


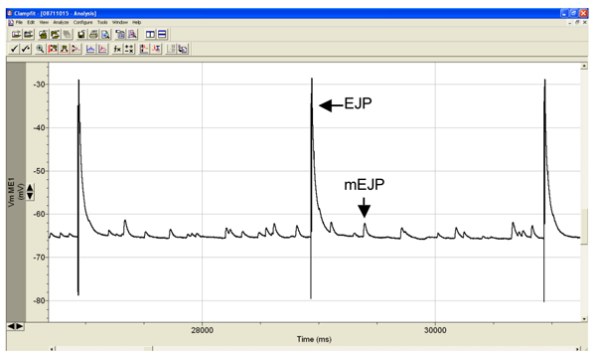
 **Figure 3.** Representative intracellular recording from muscle 6 showing the evoked EJP’s is response to electrical stimulation of the segmental nerve, and the sporadic minature endplate potentials, or mEJP’s. EJP amplitudes in muscle 6 of healthy wild-type larvae, such as Canton S, are typically around 40 mV and the mEJP amplitudes between 1-3 mV.

## Discussion

The methods described here provide a relatively quick and broad way to detect changes in synaptic function at the NMJ. The ability to perform electrophysiological recordings using intact animals in vivo, and perform genetic or pharmacological manipulations, make Drosophila an ideal animal model for investigating the physiological and genetic aspects of neurotransmission.

Since muscle cells are very large, some might prefer to add an additional step to this protocol for two electrode voltage clamp (TEVC) recording. This can be performed on the same larval preparation with the intracellular electrode in place, by positioning a current-passing electrode on the cell. Once the cell is sufficiently voltage clamped, the current response can be recorded.

Although muscle 6 is the most commonly used for electrophysiology recordings, muscles 7 and 12 can also be used.

The methods described here show the essentials of Drosophophila NMJ electrophysiology – techniques that were first described by Jan and Jan in 1976, and have since become the model system for researching synaptic physiology.
